# Association Between Healthy Lifestyle and Cognitive Function in Middle-Aged and Older Adults

**DOI:** 10.3390/healthcare13101140

**Published:** 2025-05-14

**Authors:** Rouba Khalil Naaman, Shoug Alashmali, Manar Abduljalil Bakhsh, Shomookh Ahmed Alneami, Elaf Saeed Algamdi, Ghaday Abdulwahab Al-Ghamdi, Shouq Mohammed Alqarni

**Affiliations:** 1 Department of Clinical Nutrition, Faculty of Applied Medical Sciences, King Abdulaziz University, P.O. Box 80215, Jeddah 21589, Saudi Arabia; smsaleh1@kau.edu.sa (S.A.); mabakhsh@kau.edu.sa (M.A.B.); salneami0001@stu.kau.edu.sa (S.A.A.); ealghamdi0193@stu.kau.edu.sa (E.S.A.); galghamdi0120@stu.kau.edu.sa (G.A.A.-G.); salqarni0215@stu.kau.edu.sa (S.M.A.); 2 Department of Women and Children’s Health, King’s College London, London SE1 7EH, UK

**Keywords:** cognition, healthy lifestyle, diet, elderly, MoCA, Saudi Arabia

## Abstract

**Background/Objectives**: The adherence to a healthy lifestyle is important for supporting healthy cognitive function as aging progresses. This study aimed to assess the association between a healthy lifestyle, specifically diet quality, physical activity, and smoking, and cognitive function in middle-aged and older adults. **Methods**: A cross-sectional study was conducted among participants aged 45 and older with normal cognition. Participants’ cognitive performance was assessed by the Montreal Cognitive Assessment (MoCA). Dietary intake was assessed using a 24 h dietary recall, and diet quality was assessed using the Diet Quality Index-International (DQI-I). Physical activity was assessed using the International Physical Activity Questionnaire (IPAQ). Participants were categorized as unhealthy (score ≤ 1), average (score 2), and healthy (score 3). **Results**: In this study, a total of 176 participants were recruited, 46% of them were classified as unhealthy, 40% was average, and 14% as healthy. Participants in the unhealthy group had lower performance in the naming cognitive domain compared to the other groups (*p* = 0.01). Participants in the average group scored significantly lower than the other groups in the orientation domain (*p* = 0.02). Performing moderate and high physical activity levels were significantly associated with higher scores of MoCA (*p* = 0.04) and in the naming domain (*p* = 0.02). **Conclusions**: Adopting a healthy lifestyle is associated with maintaining cognitive health among middle-aged and older adults, supporting the relevance of multidomain lifestyle interventions. Further longitudinal research is warranted to evaluate the long-term feasibility and effectiveness of lifestyle interventions in this population.

## 1. Introduction

Global life expectancy is forecasted to increase from 73 years in 2020 to 77 years by 2050 and 82 years by 2100, thus leading to a rapidly expanding elderly population, with the rate of 9.3% in 2020 increasing to 22.4% by 2100 [1]. In accordance with this global trend, Saudi Arabia is experiencing a steady increase in its elderly population, exacerbated by declining fertility rates, which has led to significant societal and economic pressures, along with increasing demands on health-care and welfare systems [2]. In addition, the elderly population faces increased incidence rates of chronic diseases, which escalate with age and constitute a significant public health concern [3]. One of the most concerning health issues is cognitive decline, which is driven by a combination of biological, psychological, and social factors, and leads to deteriorating cognitive abilities, such as memory loss and impaired decision-making, particularly in patients aged 85 years and older [4,5].

The impact of cognitive decline on health and well-being is multifaceted, including increased dependency on caregivers for daily activities, thereby affecting quality of life and autonomy [5,6]. Cognitive decline is also associated with a higher risk of comorbidities, including depression and physical health issues, which can exacerbate the overall health status of elderly individuals [5,6,7]. Furthermore, its global health and economic burdens are huge and constantly increasing. For instance, the total economic cost of dementia, a severe form of cognitive decline, was estimated at USD 1313.4 billion in 2019 for an estimated prevalence of 55.2 million people [7]. A systematic review of 47 studies revealed that the crude prevalence rate of dementia increases twofold every 5 years [8]. However, although two-thirds of dementia cases occur in low- and middle-income countries, most global expenditures are made by high-income nations [9]. This disparity may explain the recently declining incidence rates of dementia in more than 70 countries, while global trends continue to increase, highlighting the effectiveness of preventive strategies in wealthier regions [7,9].

Several techniques have been used to improve cognitive functioning, including brain exercise, securing enough sleep, meditation, and the utilization of biofeedback and neurofeedback techniques [10,11]. However, there is currently no effective cure for modifying the progression of cognitive decline and dementia. Identifying modifiable risk factors that support healthy cognitive aging has emerged as a key focus in public health research. Various lifestyle factors have been identified as potential preventive measures against cognitive decline. Among these factors, a healthy diet rich in fruits and vegetables, and physical activity are observed to be the most effective lifestyle factors for preventing cognitive decline. Physical exercise was consistently linked to improvements across various cognitive domains. Diet, particularly that adhering to the Mediterranean and Dietary Approaches to Stop Hypertension (DASH), has been shown to slow cognitive aging [12,13]. Smoking cessation and social engagement also play crucial roles in inhibiting cognitive aging, while alcohol, poor sleep quality, obesity, and prolonged sedentary behaviors have detrimental effects on cognition [12,13]. These findings support a multidomain approach to promoting cognitive health that involves physical activity, balanced nutrition, and behavioral management [14].

A few studies in Saudi Arabia have analyzed the association between cognitive decline and lifestyle factors among middle-aged and older adults; however, the available data on these factors are limited. For instance, Sulaiman et al. found that malnutrition was associated with lower Mini-Mental State Examination (MMSE) scores [15]. In another study, Alsebayel et al. analyzed various modifiable risk factors of dementia in primary care patients and reported no relationships with living status, smoking, and exercise. However, they observed a significant relationship between depression and dyslipidemia [16]. A clinical trial that assessed the effects of a 16-week walking program on frailty, cognitive performance, and quality of life was conducted among inactive older adults aged between 60 and 70 years in Saudi Arabia [17]. While the results showed improvements in physical function and performance, no notable cognitive benefits were observed, likely owing to the 12-week follow-up limitation of the study [17,18]. On the other hand, assessments of nutritional behaviors and physical activity levels among this subgroup in Saudi Arabia revealed poor and discouraging outcomes [19]. However, in spite of these challenges, tremendous efforts, such as home care programs and age-friendly health-care strategies, have been introduced to improve the well-being of the Saudi elderly population [20].

The limited scope of existing studies on cognitive decline and lifestyle factors in Saudi Arabia leaves critical associations underexplored, hindering the development of comprehensive prevention strategies to better address the increasing cognitive health challenges among middle-aged and older adults. Thus, the aim of this study was to assess the association of healthy lifestyle factors, including diet quality, physical activity, and smoking, with cognitive function in middle-aged and older adults in Saudi Arabia.

## 2. Materials and Methods

### 2.1. Design

This cross-sectional study was conducted between December 2020 and April 2023. It was approved by the Unit of the Biomedical Ethics Research Committee at King Abdulaziz University in Jeddah, Saudi Arabia (Reference No. 596-20).

### 2.2. Participants and Recruitment

The eligibility criteria for inclusion in the study were Saudi nationals aged ≥45 years, both male and female, who were residing in Jeddah and had a normal cognitive status (as assessed using the Abbreviated Mental Test (AMT-4), where a score of ≥3 out of 4 was required) [21,22]. Participants with neurological disorders or conditions that could affect their vision or hearing and those on medications that impact cognitive function were excluded. Recruitment was carried out by contacting a convenience sample of eligible participants through WhatsApp messages (Version 2.21.50), inviting them to participate in the study by providing study information. To reach a larger number of participants, four registered dietitians were contacted and asked to share the study details with their eligible contacts. Participants who agreed to participate in the study were interviewed by trained dietitians. All study participants signed an informed consent form before their inclusion.

### 2.3. Sampling

The study sample size was calculated using the Epi Info sample size calculator (Division of Health Informatics and Surveillance, Center for Surveillance, Epidemiology, and Laboratory Services, Atlanta, GA, USA). The calculation was based on the number of Saudi adults aged ≥45 years who were residing in the Makkah region [23], with an expected frequency of 20%, corresponding to the prevalence of mild cognitive impairment in Saudi Arabia [15]. The calculation considered a confidence level of 90%, a margin of error of 5%, and a design effect of 1. The required sample size was 173 participants.

### 2.4. Data Collection

A structured questionnaire was used to collect the following data.

#### 2.4.1. Sociodemographic and Medical Data

The collected sociodemographic data included age, sex, nationality, marital status, income, living situation, and educational level. In addition, the participants were asked about their history of chronic diseases, medication use, and dietary supplement use.

#### 2.4.2. Anthropometric Parameters

Body weight was measured to the nearest 0.1 kg using a calibrated scale, and height was measured to the nearest 0.1 cm using a stadiometer. Body mass index was obtained using the measured weight and height.

#### 2.4.3. Cognitive Function

Cognitive function was evaluated using the Arabic version of the Montreal Cognitive Assessment (MoCA) [24]. MoCA is a validated tool for screening for mild cognitive dysfunction in the elderly. It assesses multiple domains, including visuospatial/executive functions, naming, memory/recall, attention, language, abstraction, calculation, and orientation, with a normal cognitive status defined by a score of ≥26 out of 30.

#### 2.4.4. Lifestyle Factor Assessment

- Dietary intake

Dietary intake was assessed using a 24 h dietary recall during the interview, in which the participants reported the type and quantity of food and beverages they consumed. Detailed descriptions, portion sizes, and brand names were recorded for each meal. A second 24 h recall was conducted for a subsample of 100 participants (57.8%) on a different day, and the data were analyzed using the Automated Self-Administered 24-Hour Dietary Assessment tool (ASA24) [25]. The Diet Quality Index-International (DQI-I) [26] was used to assess diet quality, with scores ranging from 0 to 100, where a score of ≥60% indicated a good-quality diet and <60% indicated poor diet quality. The DQI-I assesses four aspects of diet: variety, adequacy, moderation, and overall balance. Variety is assessed to determine overall dietary diversity and protein sources (score range, 0–20), while adequacy is evaluated to determine the intake of key food groups and nutrients (score range, 0–40), including vegetables, fruits, grains, fiber, protein, iron, calcium, and vitamin C (based on the United States Department of Agriculture guidelines) [27]. Moderation evaluates the intake of restricted nutrients, such as total fat, cholesterol, and sodium (score range, 0–30), and overall balance evaluates macronutrient and fatty acid ratios (score range, 0–10) [26].

- Physical activity

Physical activity was assessed using the short Arabic version of the International Physical Activity Questionnaire (IPAQ) [28]. The participants reported the number of days and minutes they had engaged in walking and moderate and vigorous activities over the last 7 days. The metabolic equivalent of task (MET) was calculated for each activity, and the participants were classified as having low, moderate, or high physical activity levels, following international guidelines [29]. Participants performing moderate or high levels of physical activity were considered as having a healthy physical activity profile according to the guidelines of physical activity for older adults [29,30].

- Smoking status

The participants were asked whether they smoked, with nonsmokers considered to follow a healthy lifestyle.

### 2.5. Variables

The associations between diet quality, physical activity, smoking, and cognitive performance was assessed. A composite healthy lifestyle score (ranging from 0 to 3) was calculated, assigning 1 point for each healthy lifestyle factor. The participants were categorized as unhealthy (score, ≤1), average (score, 2), or healthy (score, 3).

### 2.6. Statistical Analysis

Statistical analysis was performed using Minitab (Version 22). Categorical variables were presented as numbers and percentages, whereas continuous variables were presented as means and standard deviations. The chi-square test and two-sample *t*-test were used to test for differences in the categorical and continuous variables, respectively. Univariate regression analysis was performed to evaluate the associations between the healthy-lifestyle groups and the *Z*-scores in the MoCA cognitive domains. A *p* value < 0.05 was considered statistically significant.

## 3. Results

### 3.1. Characteristics of the Study Participants

A total of 176 participants were recruited in this study. General characteristics of the sample by healthy-lifestyle groups are presented in Table 1. The mean age of the study population was 53.5 ± 8 years. The majority (91%) were aged between 45 and 64 years, and 68% were female. Most participants (86%) were married, and 75% had higher education. A large proportion (59%) were employed, with 56% earning more than SAR 10,000 per month. Almost half (47%) reported having a chronic disease, with 26% having heart diseases and 17% diabetes. Regarding body weight, 44% were overweight, and 34% were obese. Additionally, 53% reported using dietary supplements, and 47% were on medications. In terms of healthy lifestyle habits, 68% had poor dietary habits, 49% reported low levels of physical activity, and 24% were smokers.

Marital status was the only factor significantly associated with a healthy lifestyle score (*p* < 0.005). However, several variables, while not statistically significant, showed interesting trends. These included a tendency for a healthier lifestyle among the middle-aged elderly group, females, those with lower economic status, the unemployed, and those with a normal weight.

### 3.2. Association of Healthy Lifestyle with the MoCA Cognitive Domains

**Figure 1:**
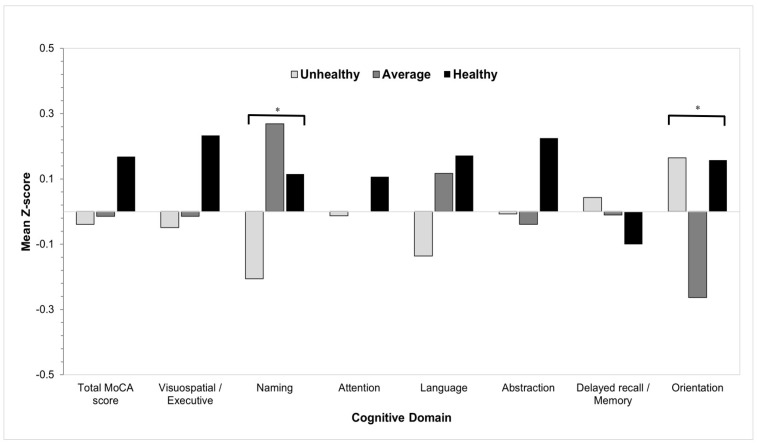
The association between healthy-lifestyle groups versus the MoCA cognitive domains scores assessed by univariate regression analysis. * *p* < 0.05.

After adjusting for age, gender, educational level, income, BMI, diabetes, and heart diseases, results showed that the unhealthy group had significantly lower scores in the naming cognitive domain compared to the average and healthy groups (*p* = 0.01) (Table 2 and Figure 1). Participants in the average group scored significantly lower than those in the unhealthy and healthy groups in the orientation domain (*p* = 0.02). No significant associations were found between the three groups in the other cognitive domains (*p* > 0.05).

### 3.3. Association of Diet Quality, Physical Activity, and Smoking with Cognitive Performance

Table 3 presents the association between diet quality, physical activity, and smoking status and cognitive performance. Participants with a good quality diet had significantly higher scores in the naming cognitive domain (*p* = 0.05). Those with moderate and high physical activity had significantly higher overall MoCA scores (*p* = 0.04) and higher scores in the naming domain (*p* = 0.02) compared to those with low physical activity. A trend was observed between high levels of physical activity and higher visuospatial/executive and language domain scores (*p* = 0.08 and *p* = 0.07, respectively). No significant associations were found between smoking status and cognitive performance (*p* > 0.05).

### 3.4. Diet Quality and Nutrient Intakes Differences in Group with Normal and Poor Cognitive Performance

While no statistically significant differences were observed in the total DQI-I score or other dimensions of diet quality (*p* > 0.05), participants with normal cognition had a relatively higher score in the adequacy aspect of the diet, with a 1.18-point difference in mean scores compared to those with poor cognition (*p* = 0.10) (Table 4).

Table 5 presents the differences in energy and nutrient intakes between the normal and poor cognition groups. The normal cognition group had significantly higher intakes of carbohydrates, calcium, magnesium, sodium, and folic acid compared to the poor cognition group (*p* < 0.05). No significant differences in energy and other nutrient intakes were found between the two groups (*p* > 0.05).

## 4. Discussion

An understanding of the association between adapting a healthy lifestyle and cognitive performance in middle-aged and older adults in Saudi Arabia is lacking. Given the increasing prevalence rate of cognitive decline in the Saudi population, identifying lifestyle factors that may contribute to cognitive health is crucial for creating preventive strategies and providing health recommendations. The results of the present study reveal that the participants who had a healthy lifestyle, including consuming a high-quality diet, performing higher levels of physical activity, and being a nonsmoker, had higher cognitive assessment scores, particularly in the naming and orientation cognitive domains.

The associations of several lifestyle factors, including diet quality [31], physical activity performance [32,33], and smoking status [34], with cognitive function have been extensively evaluated in previous studies. While most previous studies have studied single-factor effects, the present study is among the few studies that assessed single-factor effects in addition to the combined influence of the three factors on cognitive performance in middle-aged and older adult populations. When the overall association between adapting a healthy lifestyle and cognitive status was assessed in the present study, it was found that participants with an unhealthy lifestyle exhibited significantly lower performance levels in the naming cognitive domain. Additionally, the average-lifestyle group showed lower performance compared to the other groups in the orientation domain. These findings support the potential impact of adhering to a combination of the three lifestyle factors on cognitive functioning. This is consistent with a previous cross-sectional study that examined the association between six lifestyle factors with cognitive impairment among Chinese community-dwelling older adults [35]. Similarly, a cohort study conducted in the United Kingdom assessed the relationship between adherence to healthy lifestyle practices and cognitive impairment among middle-aged men [36]. Both studies reported that adhering to a greater number of healthy lifestyle factors was associated with better cognitive performance [35,36]. Although the number of lifestyle factors and scoring components varied across the studies, diet quality, physical activity, and smoking were consistently considered lifestyle factors in both the current and previous studies.

Of all the cognition domains evaluated in this study using MoCA, naming and orientation were likely to be more influenced by lifestyle changes. The specificity of these domains is a nuanced area of research that highlights the differential sensitivity of cognitive domains to various lifestyle interventions. Evidence suggests that certain cognitive domains are more susceptible to lifestyle modifications than others [37]. Results from the Finnish Geriatric Intervention Study to Prevent Cognitive Impairment and Disability (FINGER) trial demonstrated that lifestyle interventions, particularly those targeting diet, vascular risk control, and cognitive activity, significantly impacted executive functions and global cognition [37]. Conversely, another controlled trial by Küster et al. showed a positive association between memory and lifestyle changes, whereas attention and executive functions did not exhibit similar benefits [38]. Thus, naming and orientation are particularly sensitive owing to their reliance on a combination of memory recall and spatial awareness, both of which can be influenced by lifestyle factors.

The present study reported a positive influence of good diet quality on cognition performance, notably the naming domain. The relationship between diet quality and cognitive preservation has been extensively studied through various longitudinal studies and clinical trials. Evidence suggests that adherence to the Mediterranean diet, which emphasizes increased intakes of fruits, vegetables, whole grains, and healthy fats, has been consistently linked with better cognitive functioning and a slower rate of cognitive decline, along with a reduced risk of Alzheimer’s disease in older adults [39]. Similar associations were found with adherence to neuroprotective diets, such as the Mediterranean–DASH Intervention for Neurodegenerative Delay diet, a hybrid of the Mediterranean and DASH diets that emphasizes foods such as leafy greens, berries, nuts, olive oil, and fish, while limiting red meat, butter, and sweets [40]. The positive effect of adhering to a high-quality diet on cognition may be explained by the fact that those who adopt a healthy dietary pattern are expected to consume adequate levels to meet their nutrient requirements. In line with this, the present study found that the participants with normal cognition consumed an adequate diet and had relatively higher scores in this aspect of the DQI-I. This highlights the importance of consuming a well-balanced diet that is rich in all needed nutrients for preserving cognitive health and function in this population.

Although the overall reported intake of calcium, magnesium, and folic acid among all participants in the current study was lower than the recommended daily requirements [41], a positive association was found between cognitive performance and increased intake of those nutrients. This association may be explained by the role of these nutrients in preserving cognitive function [42]. Adequate calcium levels facilitate effective neural communication, supporting cognitive tasks [43]. Magnesium, on the other hand, is involved in several vital mechanisms within the central nervous system and plays a role in maintaining the integrity of blood–brain barrier [44,45]. Folic acid is important for DNA synthesis and repair, which contributes to cognitive preservation [46]. Consistent with the current study findings, several other studies examined these relationships and provided evidence that adequate intake of dietary calcium [47], magnesium [48], and folic acid [49] may help mitigate cognitive decline. However, prior studies primarily involved older adults and used different tools to assess cognition. Regarding sodium intake and cognitive performance, mixed findings were reported [50,51,52]. In this study, participants with higher MoCA scores had significantly higher sodium intake. Similarly, an American study found that higher dietary sodium intake was associated with better cognitive function in community-dwelling older adults [51]. While that study assessed sodium intake using a food frequency questionnaire, the mean of the quartile with the highest sodium intake (2628 mg/day) was within the range observed in the current study (2787 mg/day) [51]. These findings suggest that maintaining an adequate dietary balance is crucial for cognitive health, reinforcing the idea that optimal mineral intake can influence cognitive outcomes. Therefore, future research is needed to determine the optimal amounts of these nutrients, including sodium, required to produce significant effects on cognitive performance.

The association between performing physical activity and better cognitive functioning has been previously reported in young adults [53] and in middle-aged and older adults [54]. In line with previous study findings, this study found that among the lifestyle factors, physical activity appeared to have a positive effect on cognitive status. The participants who performed physical activity at moderate and high levels had significantly higher overall MoCA scores, particularly in the naming domain, than those who performed physical activity at low levels. Moreover, the present study reported a trend between higher physical activity performance levels and higher scores in the visuospatial/executive and language domains. This is consistent with findings reported in a systematic review and meta-analysis of longitudinal studies highlighted that performing physical activity was associated with a lower risk of deteriorated cognition and dementia across various populations, emphasizing the importance of encouraging physical activity regardless of its direct relationship with cognitive outcomes [55]. Furthermore, the results of Cheema’s research indicate that combining cognitive training with aerobic exercise yields even greater improvements in cognitive functioning, which suggests that integrated approaches may be particularly effective [56].

The mechanisms through which physical activity exerts its cognitive benefits are multifaceted. Exercise is known to promote neurogenesis, enhance synaptic plasticity, and improve cerebral blood flow, all of which contribute to better cognitive health [57]. The findings of Rabin et al. indicate that greater physical activity correlates with lower levels of β-amyloid, a biomarker associated with Alzheimer’s disease, which suggests that physical activity may play a protective role against neurodegeneration [58].

The findings of this study provide valuable insights for public health action, particularly in Saudi Arabia, where efforts have already been made to enhance the well-being of the elderly population through home care programs and age-friendly health-care strategies [20]. The Saudi Ministry of Health published a protocol in 2023 designed to provide recommendations for the prevention of cognitive impairment [59]. The protocol emphasized the significant influence of modifiable risk factors on mental health including physical activity, dietary intake, and smoking. However, additional measures are required to address cognitive decline from a preventive perspective. Clinicians should be educated to prioritize lifestyle interventions, especially physical activity, as it has the strongest effect on cognitive preservation. Integrating systematic diet counseling into elderly care can further mitigate cognitive decline. Public health efforts should raise awareness of the importance of healthy lifestyles in promoting cognitive health. Expanding programs in community centers that promote physical activity, nutrition, and social engagement is essential, particularly for the elderly aged >65 years. Policies should promote neuroprotective diets, such as the Mediterranean and MIND diets, while emphasizing nutrient balance. Public campaigns should address both nutrient adequacy and the risks of excessive intake of nutrients, especially calcium. More longitudinal studies are needed to assess the causal relationships between lifestyle and cognitive health in Saudi Arabia. To examine for more robust and long-lasting effects, future research should not only focus on middle-aged and older adults aged >65 but also consider younger populations. In addition, integrated approaches combining diet, exercise, and cognitive training should be explored for comprehensive cognitive health benefits.

This study was limited mainly by its cross-sectional design, which limited the ability to infer causality between lifestyle factors and cognitive outcomes, and the potential for recall bias, especially in dietary data collection. Moreover, the use of 24 h dietary recall introduces recall bias and may not accurately represent typical intake, limiting the validity and generalizability of the findings related to diet quality. While the ASA24 is a valid tool for analyzing dietary intake, it has not yet been validated for Arabic-speaking populations. The low representation of individuals aged ≥65 (only 9% of the sample) limits the ability to generalize findings to the highest-risk population. MoCA, while validated in Arabic, may yield inconsistencies if not administered uniformly across participants. Finally, relying on a single measurement per domain (e.g., short IPAQ) may underestimate the complexity of the behaviors assessed.

## 5. Conclusions

The present study emphasizes the importance of diet and physical activity in maintaining cognitive health in middle-aged and older Saudi adults. It highlights the potential role of specific nutrients, which may be particularly relevant to the local population. These findings support the use of multidomain lifestyle interventions that incorporate population-wide awareness, health-care guidelines, and food regulations to enhance cognitive resilience and prevent cognitive decline.

Despite the limitations of its cross-sectional design, this study underscores the need for further longitudinal research that includes a larger sample of older adults to fully elucidate the long-term impacts of lifestyle interventions on cognitive performance in the Saudi older adults. In addition, future research should aim to identify the key nutrient deficits contributing to cognitive decline in this population and assess the effectiveness of targeted dietary interventions in mitigating these deficits and improving cognitive health outcomes.

## Figures and Tables

**Table 1:** Participants’ general characteristics (*n* = 176).

Variables		Healthy-Lifestyle Groups			*p* Value
	Total (*n* = 176)	Unhealthy (*n* = 81)	Average (*n* = 70)	Healthy (*n* = 25)	
**Age (years)**					
Mean ± SD	53.5 ± 8	54 ± 8	53 ± 8	53 ± 8	0.46
45–64	161 (91)	75 (93%)	62 (87%)	24 (96%)	
≥65	15 (9)	6 (7%)	8 (11%)	1 (4%)	
**Gender**					
Male	56 (32)	31 (38)	18 (26)	7 (28)	0.23
Female	120 (68)	50 (62)	52 (74)	18 (72)	
**Marital status**					
Married	151 (86)	61 (75)	65 (93)	25 (100)	0.005
Single	10 (6)	9 (11)	1 (1)	0 (0.0)	
Widowed	15 (8)	11 (14)	4 (6)	0 (0.0)	
**Educational level**					
School education or less	44 (25)	23 (28)	14 (20)	7 (28)	0.46
Higher education	132 (75)	58 (72)	56 (80)	18 (72)	
**Work status**					
Employed	104 (59)	51 (64)	42 (60)	11 (44)	0.51
Unemployed	34 (19)	15 (18)	12 (17)	7 (28)	
Retired	38 (22)	15 (18)	16 (23)	7 (28)	
**Income (SR) ^1^**					
<4000	25 (14)	10 (12)	10 (15)	5 (20)	0.44
4000–7000	20 (12)	6 (8)	11 (16)	3 (12)	
8000–10,000	31 (18)	13 (16)	12 (18)	6 (24)	
>10,000	98 (56)	52 (64)	35 (51)	11 (44)	
**Chronic diseases**					
No diseases	94 (53)	40 (49)	38 (54)	16 (64)	0.43
Diabetes	30 (17)	12 (15)	13 (19)	5 (20)	0.75
Heart diseases	46 (26)	23 (28)	20 (29)	3 (12)	0.22
Respiratory diseases	10 (6)	6 (7)	4 (6)	0 (0)	0.37
Thyroid gland disorders	22 (12)	10 (12)	10 (14)	2 (8)	0.71
**Medications**					
Yes	82 (47)	42 (52)	30 (43)	10 (40)	0.42
No	94 (53)	39 (48)	40 (57)	15 (60)	
**Dietary supplement**					
Yes ^2^	94 (53)	39 (48)	41 (59)	14 (56)	0.48
No	82 (47)	42 (52)	29 (41)	11 (44)	
**BMI category**					
Under weight	2 (1)	1 (1)	1 (1)	0 (0)	0.86
Normal weight	37 (21)	17 (21)	13 (19)	7 (28)	
Overweight	78 (44)	38 (47)	30 (43)	10 (40)	
Obese	59 (34)	25 (31)	26 (37)	8 (32)	
**Diet quality**					
Poor quality	120 (68)	73 (90)	47 (67)	0	<0.001
Good quality	56 (32)	8 (10)	23 (33)	25 (100)	
**Physical activity**					
Low intensity (<600 METs)	86 (49)	68 (84)	18 (26)	0 (0)	<0.001
Moderate intensity (at least 600 METs)	69 (39)	9 (11)	42 (60)	18 (72)	
High intensity (at least 3000 METs)	21 (12)	4 (5)	10 (14)	7 (28)	
**Smoking**					
Yes	42 (24)	37 (46)	5 (7)	0 (0)	<0.001
No	134 (76)	44 (54)	65 (93)	25 (100)	

SD, standard deviation; SR, Saudi Riyals; BMI, body mass index; MET, metabolic equivalent of task. Data are presented as number and percentage unless otherwise stated. The difference between the three groups was assessed by chi-square test. ^1^ Data were missing for 2 participants. ^2^ Supplements include multivitamin, vitamin D, vitamin B1-B6-B12, vitamin E, vitamin C, folic acid, calcium, iron, magnesium, selenium, zinc, omega-3-6-9 fatty acids, and bio-garlic.

**Table 2:** The association between healthy lifestyle versus the MoCA cognitive domains scores assessed by univariate regression analysis.

Moca Domains	Unadjusted Healthy-Lifestyle Groups			Adjusted Healthy-Lifestyle Groups		
	Average Coefficient	Healthy Coefficient	*p*-Value	Average Coefficient	Healthy Coefficient	*p*-Value ^1^
Overall MoCA score	0.025	0.207	0.65	0.008	0.256	0.43
Visuospatial/executive	0.035	0.282	0.46	−0.005	0.326	0.28
Naming	0.475	0.321	0.01	0.484	0.347	0.01
Attention	0.013	0.020	0.86	0.043	0.269	0.47
Language	0.252	0.308	0.20	0.207	0.294	0.27
Abstraction	−0.032	0.232	0.51	0.012	0.334	0.31
Delayed recall/memory	−0.053	−0.143	0.82	−0.126	−0.204	0.55
Orientation	−0.429	−0.008	0.02	−0.472	−0.067	0.01

MoCA, Montreal Cognitive Assessment. ^1^ Adjusted for age, gender, educational level, income, BMI, diabetes, and heart diseases.

**Table 3:** The association of diet quality, physical activity, and smoking status with the MoCA cognitive domains scores.

Moca Domains	Diet Quality			Physical Activity				Smoking		
	Poor	Good	*p* Value ^1^	Low	Moderate	High	*p* Value ^2^	Yes	No	*p* Value ^1^
Overall MoCA score	0.01 ± 1.07	0.02 ± 0.83	0.82	−0.19 ± 1.11	0.20 ± 0.85	0.10 ± 0.87	0.04	0.12 ± 0.99	−0.04 ± 1.00	0.38
Visuospatial/executive	−0.01 ± 1.06	0.03 ± 0.88	0.78	−0.16 ± 1.11	0.15 ± 0.83	0.23 ± 0.99	0.08	0.11 ± 0.92	−0.03 ± 1.03	0.41
Naming	−0.05 ± 1.18	0.20 ± 0.51	0.05	−0.18 ± 1.37	0.27 ± 0.00	0.08 ± 0.84	0.02	0.18 ± 0.59	−0.02 ± 1.12	0.14
Attention	0.01 ± 1.03	0.01 ± 0.94	0.97	−0.06 ± 1.11	0.11 ± 0.89	−0.01 ± 0.88	0.57	0.10 ± 0.93	−0.02 ± 1.02	0.49
Language	−0.05 ± 0.98	0.14 ± 1.06	0.26	−0.17 ± 1.00	0.17 ± 0.99	0.20 ± 0.98	0.07	−0.13 ±1.06	0.05 ± 0.99	0.33
Abstraction	−0.03 ± 1.07	0.11 ± 0.83	0.32	−0.07 ± 1.07	0.13 ± 0.81	−0.05 ± 1.28	0.43	0.07 ± 1.04	−0.00 ± 0.99	0.69
Delayed recall/memory	0.05 ± 1.01	−0.10 ± 1.04	0.38	−0.09 ± 1.02	0.09 ± 1.04	0.06 ± 0.95	0.52	0.06 ± 0.98	−0.02 ± 1.03	0.63
Orientation	−0.00 ± 1.10	−0.01 ± 0.82	0.90	0.05 ± 0.83	−0.00 ± 1.12	−0.24 ± 1.35	0.51	0.07 ± 0.90	−0.03 ± 1.05	0.52

MoCA, Montreal Cognitive Assessment. Data are presented as means ± standard deviations. ^1^ The difference between the three groups was assessed by a 2-sample *t*-test. ^2^ The difference between the three groups was assessed by one-way ANOVA test.

**Table 4:** Diet quality aspects of normal cognition and poor cognition groups.

Diet Quality Aspects	Total (*n* = 176)	Normal Cognition (*n* = 103)	Poor Cognition (*n* = 73)	*p*-Value ^1^
DQI-I total score	54.92 ± 8.94	55.11 ± 9.12	54.66 ± 8.74	0.74
Variety	14.08 ± 3.05	14.19 ± 3.15	13.92 ± 2.92	0.55
Adequacy	22.55 ± 4.77	23.05 ± 4.78	21.87 ± 4.70	0.10
Moderation	13.88 ± 5.27	13.48 ± 5.15	14.44 ± 5.42	0.23
Overall balance	4.40 ± 2.92	4.38 ± 2.84	4.44 ± 3.04	0.89

DQI-I, Diet Quality Index-International. Data are presented as means ± standard deviations. ^1^ The difference between the groups was assessed by a 2-sample *t*-test.

**Table 5:** Energy and nutrient intake differences between normal cognition and poor cognition groups.

Nutrients	Total (*n* = 176)	Normal Cognition (*n* = 103)	Poor Cognition (*n* = 73)	*p* Value ^1^
Energy (kcal)	1595 ± 632.7	1663 ± 619.3	1498 ± 643	0.09
Carbohydrate (g)	182.8 ± 77.8	193.5 ± 80.2	167.6 ± 72.1	0.02
Protein (g)	71 ± 33.9	72.8 ± 33.9	68.6 ± 33.9	0.42
Total fat (g)	65.9 ± 30.5	67.9 ± 28.1	63.1 ± 33.6	0.32
Saturated fat (g)	20.8 ± 10.6	21.2 ± 10.1	20.1 ± 11.4	0.50
Fiber (g)	13.4 ± 7.2	14.0 ± 6.5	12.4 ± 7.9	0.15
Calcium (mg)	671.3 ± 413.8	719.5 ± 461.2	603.4 ± 326.8	0.05
Iron (mg)	10.6 ± 5.4	11.1 ± 5.6	9.9 ± 4.9	0.13
Zinc (mg)	8.4 ± 5	8.5 ± 4.8	8.3 ± 5.3	0.82
Magnesium (mg)	227.4 ± 97.3	239.7 ± 90.7	210 ± 104.1	0.05
Selenium (mcg)	114 ± 65.5	120.1 ± 70.5	105.5 ± 57.2	0.13
Sodium (mg)	2623.3 ± 1257.7	2787 ± 1275	2392 ± 1203	0.03
Potassium (mg)	1961.8 ± 789.6	2033 ±764.8	1861.4 ± 818	0.16
Vitamin B12 (mcg)	5.7 ± 10.7	5.2 ± 8.4	6.5 ± 13.3	0.48
Folic acid (mcg)	330.8 ±153.6	350 ± 156.6	303.7 ± 146	0.04
Vitamin D (IU)	241.2 ± 325.1	238.5 ± 288.6	245.1 ± 372.7	0.89
Vitamin C (mg)	53.8 ± 57.4	57.6 ± 54	48.4 ± 61.8	0.30

kcal, kilocalorie; g, gram; mg, milligram; mcg, microgram; IU, international unit. Data are presented as means ± standard deviations. ^1^ The difference between the groups was assessed by a 2-sample *t*-test.

## Data Availability

The raw data supporting the conclusions of this article will be made available by the authors on request.

## Abbreviations

The following abbreviations are used in this manuscript:

MoCA	Montreal Cognitive Assessment
DQI-I	Diet Quality Index-International
IPAQ	International Physical Activity Questionnaire
DASH	Dietary Approaches to Stop Hypertension
MMSE	Mini-Mental State Examination
AMT-4	Abbreviated Mental Test
BMI	Body mass index
ASA-24	Automated Self-Administered Dietary Assessment tool
MET	The metabolic equivalent of task
DHA	Docosahexaenoic acid
